# A New Hybrid Model FPA-SVM Considering Cointegration for Particular Matter Concentration Forecasting: A Case Study of Kunming and Yuxi, China

**DOI:** 10.1155/2017/2843651

**Published:** 2017-08-28

**Authors:** Weide Li, Demeng Kong, Jinran Wu

**Affiliations:** ^1^School of Mathematics and Statistics, Lanzhou University, Lanzhou, Gansu 730000, China; ^2^North Automatic Control Technology Research Institute, Taiyuan, Shanxi 030006, China

## Abstract

Air pollution in China is becoming more serious especially for the particular matter (PM) because of rapid economic growth and fast expansion of urbanization. To solve the growing environment problems, daily PM2.5 and PM10 concentration data form January 1, 2015, to August 23, 2016, in Kunming and Yuxi (two important cities in Yunnan Province, China) are used to present a new hybrid model CI-FPA-SVM to forecast air PM2.5 and PM10 concentration in this paper. The proposed model involves two parts. Firstly, due to its deficiency to assess the possible correlation between different variables, the cointegration theory is introduced to get the input-output relationship and then obtain the nonlinear dynamical system with support vector machine (SVM), in which the parameters c and g are optimized by flower pollination algorithm (FPA). Six benchmark models, including FPA-SVM, CI-SVM, CI-GA-SVM, CI-PSO-SVM, CI-FPA-NN, and multiple linear regression model, are considered to verify the superiority of the proposed hybrid model. The empirical study results demonstrate that the proposed model CI-FPA-SVM is remarkably superior to all considered benchmark models for its high prediction accuracy, and the application of the model for forecasting can give effective monitoring and management of further air quality.

## 1. Introduction

Air pollution has a great impact on humans and environment [1, 2]. The information on meteorological pollution, caused by CO, NO, NO_2_, SO_2_, O_3_, and particulate matter (PM_2.5_ and PM_10_), is urgent due to the harmful effects on human health [3]. Especially in recent years, regions of China have suffered the hazy weather including Jianghuai, North China, Huanghuai, south of the Yangtze River, and other areas. The affected regions are about 25% of the country, and the affected population is above six hundred million [4]. Furthermore, the hazy weather is harmful to the respiratory and cardiovascular system of human which would induce chronic disease and cancer. In addition, it would affect mental and reproductive health. And related studies found that extreme particulate matter (PM_2.5_ and PM_10_) was one of the main factors of hazy weather [5]. So it is urgent to monitor the particulate matter and its forecasting is an important work. In view of this situation, this paper introduces a new hybrid model to forecast the daily particulate matter of Kunming and Yuxi, China.

In recent period, there are lots of researchers concentrating on the technique of predicting the PM concentration. The extreme particulate matter is an open, nonlinear, dynamic, and complex system. So it is difficult to derive an accurate formula to predict the value of PM. Fortunately, a data-driven, empirically based or “black-box” modeling approach which is designed to identify relationships between input and output without considering the mechanism of generating particulate matter can be employed to predict the PM concentration. With the development of artificial intelligence, machine learning techniques such as ANN and SVM have been applied into the time series of air pollution matter. Grivas and Chaloulakou provided reliable predictions of PM_10_ hourly concentrations by evaluating the potential of various developed neural network models [6]. Cai et al. applied artificial network to predict hourly air pollutant concentrations of Guangzhou, China [7]. Caselli et al. developed the back-propagation neural network to predict the daily PM_10_ concentration before 1, 2, and 3 days [8]. De Gennaro et al. developed an artificial neural network (ANN) to forecast PM_10_ daily concentration in two contrasted environments in NE Spain [9]. Ding et al. predicted air pollutant concentration using a feedforward neural network inspired by the mechanism of the human brain [10]. Meanwhile, the method of support vector machine is widely employed in predicting the air pollutant concentrations. Suárez Sánchez et al. proposed a regression model of air quality by using the support vector machine (SVM) technique in the Aviles urban area (Spain) at local scale [11]. García Nieto et al. presented a method of daily air pollution modeling by using support vector machine (SVM) technique in Oviedo urban area (Northern Spain) at local scale [12]. But it is difficult for one single machine learning algorithm to achieve high precise prediction [13]. So researchers combined different algorithms to get hybrid models to forecast the air pollution matter (CO, NO, NO_2_, SO_2_, O_3_, and particulate matter). Díaz-Robles et al. proposed a hybrid model combining ARIMA and ANN to improve forecast accuracy for the air quality of Temuco, Chile [14]. Feng et al. used artificial neural network to predict ozone concentration on single site with a better forecast accuracy in huge data set condition [15]. Fu et al. introduced a feedforward neural network with rolling mechanism and grey model to forecast air PM_2.5_ and PM_10_ concentrations in Hangzhou, Shanghai, and Nanjing, China [16]. Niu et al. introduced a hybrid model based on CCEMD, GWO, and SVM for daily PM_2.5_ concentration forecasting in Harbin and Chongqing, China [4]. Xu et al. proposed a hybrid model named ICEEMD-WOA-SVM for forecasting major pollutants (CO, NO, NO_2_, SO_2_, O_3_, and particulate matter) in Harbin, Chongqing, and Taiyuan, China [17]. Inspired by above researches, this paper proposes a new hybrid model with different algorithms to improve the accuracy of prediction.

As the traditional methods, many researchers established the models only using one time series. So these models may reduce the accuracy of the prediction with using insufficient information. Fortunately, Engle and Granger provided the cointegration theory to overcome the problems of nonstationarity of the time series and deal with the “spurious regression” [18]. And the forecast based on cointegration theory can put two or more sequences into the models and enhance the performance of the models. Because of its great effect, the theory has been studied in economics extensively during the past decades. Nevertheless, this theory started applying to the engineering research. Using the cointegration theory, Belloumi examined the causal relationship between per capita energy consumption and per capita gross domestic product for Tunisia during the 1971–2014 period [19]. Shahbaz et al. reexamined the relationship between electricity consumption, economic growth, and employment in Portugal using the cointegration [20]. Jahangir Alam et al. investigated the possible existence of dynamic causality between energy consumption, electricity consumption, carbon emissions, and economic growth in Bangladesh [21]. Saboori et al. established a long run as well as causal relationship between economic growth and carbon dioxide (CO_2_) emissions for Malaysia [22]. Dogan analyzed the short and long run estimates as well as the causality relationships between economic growth, electricity consumption from renewable sources, and electricity consumption from nonrenewable sources for Turkey in a multivariate model wherein capital and labor are included as additional variables [23]. In the study of hydrology, Zhang et al. introduced CI to reveal the long-term balance relationship and short-term fluctuations of the original and decomposed runoff and sediment load time series [24]. In meteorology, de Cian et al. presented an empirical study of the relationship between residential energy demand and temperature [25]. For these reasons, this paper tries to make use of the cointegration theory to find the causal relationship of PM_2.5_ and PM_10_ of Kunming and Yuxi.

In machine learning, support vector machine (SVM) has greater performance to depict nonlinear relationship. But the accuracy of SVM depends on two parameters and the optimized methods for selecting the parameters are complex and changeable. Hu et al. proposed a hybrid forecasting approach that consists of the empirical wavelet transform, coupled simulated annealing, and least square support vector machine for enhancing the accuracy of short-term wind speed forecasting [26]. Zhang et al. built a predictive model based on support vector regression and differential evolution algorithm to forecast the electricity load [27]. Liang et al. proposed a hybrid model based on wavelet transform and least squares support vector machine, which is optimized by an improved cuckoo search to predict the short-term electric load [28]. Wu and Peng built a novel hybrid approach for wind power generation forecasting in the light of cloud-based evolutionary algorithm and least squares support vector machine [29]. Santamaría-Bonfil et al. proposed a hybrid methodology based on support vector regression and genetic algorithm for wind speed forecasting [30]. W. Sun and J. Sun presented a novel hybrid model based on least squares support vector machine optimized by cuckoo search to monitor and control the PM_2.5_ concentration [31]. Sreekumar et al. presented three forecasting models, namely, three-day trained support vector regression model and parameter optimized SVR using genetic algorithm and that using particle swarm optimization in the fields of power system [32]. This paper introduces a new optimized method using flower pollination algorithm to obtain the suitable parameters for support vector regression, and this algorithm is more efficient than traditional methods such as GA and PSO [33].

Targeting at improving the predictive accuracy of PM_2.5_ and PM_10_ concentration, a hybrid model based on cointegration theory (CI), support vector machine (SVM), and flower pollination algorithm (FPA) is established. Firstly, the cointegration theory is utilized to get the causal relationship among four particular matter sequences of Kunming and Yuxi. Then the SVM technique optimized by FPA which can achieve a balance between exploration and exploitation is built to forecast particular matter (PM_2.5_ and PM_10_ concentrations) [33]. The data sets of particular matter from two cities (Kunming and Yuxi) in Yunnan Province are collected to evaluate the effectiveness of the proposed model. The remaining part of the article is organized as follows.  Section 2 mainly introduces the techniques of cointegration theory, support vector machine, and flower pollination algorithm. Next, the data of study areas, evaluation criteria, and the results of proposed hybrid model are introduced in Section 3. At last, the conclusion and future work are displayed in Section 4.

## 2. Mathematical Methods

### 2.1. Cointegration Theory (CI)

The cointegration theory is proposed by Engle and Granger to overcome the “spurious regression” of time series [18]. Cointegration mainly depicts the long-term balance relationships among nonstationary time series [24]. If a nonstationary time series is stationary after the *d* times differencing, the time series is said to be integrated of order *d*, represented as *I*(*d*). Apparently, *I*(0) is the stationary time series.

The Augment Dickey-Fuller (ADF) test is one of the most popular tests to determine the stationarity of variable series [34]. The ADF test depends on the flowing regression formula:

(1) $$\begin{array}{c} \Delta y_{t}=\alpha +\beta \cdot t+\delta \cdot y_{t-1}+\overset{p}{\underset{i=1}{\sum }}\xi_{i}\cdot \Delta y_{t-i}+\varepsilon_{t}, \end{array}$$

where *α* is the constant term; *β*, *δ*, *ξ*_*i*_ are the parameters; Δ*y*_*t*_ is the first differencing of *y*_*t*_; *t* is the time; and *ε*_*t*_ is the white noise term. Meanwhile, the lag length *p* is determined by the AIC and SC.

Engle and Granger proposed E-G test to examine the cointegration between two time series [18]. Firstly, the test establishes a regression model of the data by OLS and obtains the residues *ε*_*t*_. Then, it tries to verify the residues time series using the ADF test. If the residue is stationary, the two time series have a casual relationship on short and long run.

The Johansen test is proposed by Soren Johansen to test cointegration of several time series of *I*(*d*) [35]. The test permits more than one cointegrating relationship. There are two types of Johansen test (trace and eigenvalue). The null hypothesis for the trace and eigenvalue tests is that the number of cointegration vectors is *x* < *k* versus the alternative where *x* = *k*. Both the Johansen tests are based on the vector autoregressive model.

### 2.2. Support Vector Machine (SVM)

The support vector machine is a popular technique and its fundamental theory are introduced by Vapnik [36]. One of the advantages of SVM is minimization of structural risks, which minimize the upper-bound generalization error rather than the local training error [37]. The SVM purses the best trade-off between the model's empirical error and the model complexity [30]. The regression formula is defined as

(2) $$\begin{array}{c} f\left(\;x\;\right)=\overset{N}{\underset{i=1}{\sum }}\omega_{i}\cdot \phi_{i}\left(\;x\;\right)+b, \end{array}$$

where *b* is the bias term; *ϕ*_*i*_(*x*) is the feature. And *ω*_*i*_ of formula is optimized as

(3) max⁡ ωα,α∗=−12∑i,j=1mαi−αi∗αj−αj∗φxi,φxj−ε∑i=1mαi∗+αi+∑i=1mαi∗−αiyi,s.t. ∑i=1mαi−αi∗=0, 0≤αi≤C,∀i=1,…,m, 0≤αi∗≤C,∀i=1,…,m,

where *C* is the complexity penalization term, and *α*, *α*^*∗*^ correspond to the dual variables for the active constraints [38].

The technique converts nonlinear problem into linear problem using the kernel function *k*(*x*_*i*_, *x*). In this paper, the RBF is adopted, which can be expressed by

(4) $$\begin{array}{c} k\left(\;x_{i},x\;\right)=\exp\left\{\;-\frac{\left\|x-x_{i}\right\|}{2\sigma_{2}}\;\right\}. \end{array}$$

Finally, the nonlinear formula can be obtained by

(5) $$\begin{array}{c} f\left(\;x\;\right)=\overset{N}{\underset{i=1}{\sum }}\left(\;\alpha_{i}-\alpha_{i}^{*}\;\right)\cdot k\left(\;x_{i},x\;\right)+b. \end{array}$$

### 2.3. Flower Pollination Algorithm (FPA)

The novel swarm intelligence (SI) technique of FPA is first proposed by Yang [33]. Flower pollination is an intriguing process in the natural word. Its evolutionary characteristics can be used to design new algorithms.

The main purpose of a flower is ultimately reproduction via pollination. Pollination can take two major forms: abiotic and biotic. About 90% of flowering plants belong to biotic pollination; that is, pollen is transferred by a pollinator such as insects and animals. About 10% of pollination takes abiotic form which does not require any pollinators. The flower constancy may have evolutionary advantages because this will maximize the transfer of flower pollen to the same or conspecific plants, thus maximizing the reproduction of the same flower species [33].

Pollination can be achieved by self-pollination or cross-pollination. Cross-pollination, or allogamy, means pollination can occur from pollen of a flower of a different plant, while self-pollination is the fertilization of one flower from pollen of the same flower or different flowers of the same plant. Biotic cross-pollination may occur at long distance, and the pollinators can fly a long distance, which is considered as the global pollination. The algorithm idealizes the characteristics of pollination process, flower constancy, and pollinator behavior as the following rules:

1. Biotic and cross-pollination are considered as global pollination process with pollen-carrying pollinators performing Levy flights.
2. Abiotic and self-pollination are considered as local pollination.
3. Flower constancy can be considered as the reproduction probability which is proportional to the similarity of two involved flowers.
4. Local pollination and global pollination are controlled by a switch probability *p* ∈ [0,1]. Due to the physical proximity and other factors such as wind, local pollination can have a significant faction *p* in the overall pollination activities.

**Figure 1:**
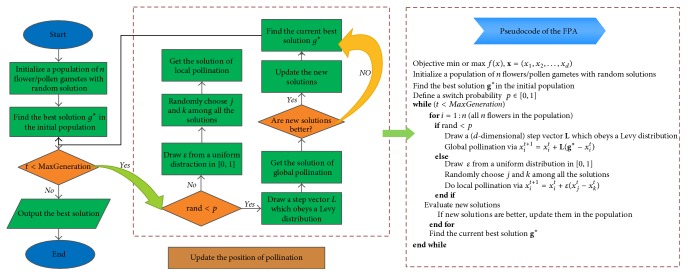
The process and pseudocode of FPA.

There are two key steps in the algorithm, the global pollination and local pollination. In the global pollination step, pollen can travel over a long distance because insects can fly and move on a longer range. The first rule plus flower constancy can be represented mathematically as

(6) $$\begin{array}{c} x_{i}^{t+1}=x_{i}^{t}+L\cdot \left(\;x_{i}^{t}-g_{*}\;\right), \end{array}$$

where *x*_*i*_^*t*^ is the pollen *i* at iteration *t*, and *g*_*∗*_ is the current best solution found among all solutions at the current iteration. The parameter *L* is the strength of pollination which drew from a Levy distribution (*λ* = 1.5). The local pollination (Rule  2) and flower constancy can be represented as

(7) $$\begin{array}{c} x_{i}^{t+1}=x_{i}^{t}+\varepsilon \cdot \left(\;x_{j}^{t}-x_{k}^{t}\;\right), \end{array}$$

where *x*_*j*_^*t*^ and *x*_*k*_^*t*^ are random pollen from the different flowers of the same plant species; *ε* is from a uniform distribution in [0,1]. And *p* = 0.8 works better for most applications from lots of simulations. The flower pollination algorithm (FPA) is presented in Figure 1.

### 2.4. The Hybrid Model CI-FPA-SVM

**Figure 2:**
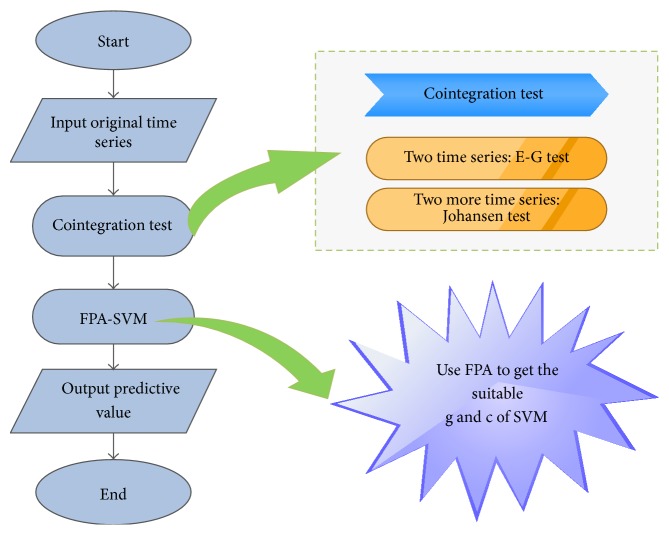
The basic structure of CI-FPA-SVM.

In this section, the proposed novel hybrid model CI-FPA-SVM is described in detail (Figure 2). First, we obtain the casual relationship among the four particular matter times series by CI with unit root test and cointegration test. Then, the nonlinear model between the input and target is built by SVM which is optimized by FPA. Finally, the prediction of PM is obtained by the proposed hybrid model. The structure of the proposed hybrid model is illustrated in Figure 2.

## 3. Empirical Study

### 3.1. Study Areas Description

**Figure 3:**
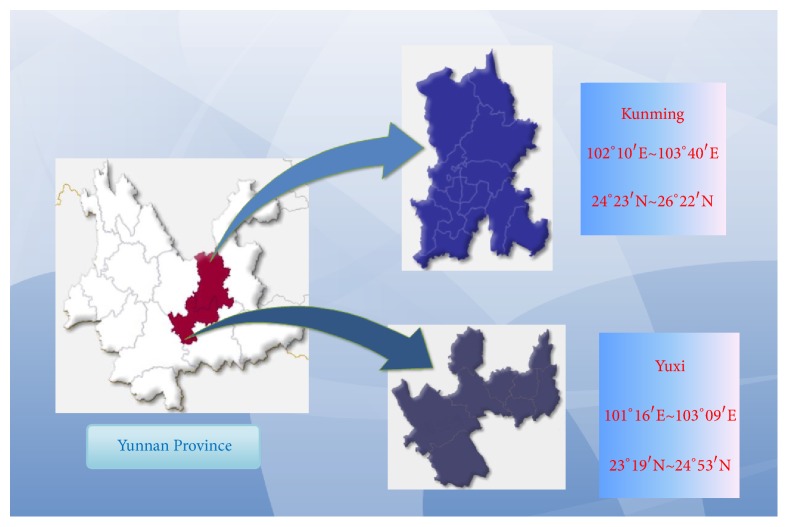
The locations of study areas.

To verify the effectiveness of the proposed hybrid model, Kunming and Yuxi are collected as the study areas (Figure 3). The detailed information of the study areas is as follows.

Kunming is the capital and largest city in Yunnan Province, Southwest China, with a population of 6.677 million in 2016. It is located between north latitude of 24°23′ and 26°22′N and east longitude of 102°10′ and 103°40′E, with a total area of 21,600 square kilometers. This city is situated in a fertile lake basin on the northern shore of the Lake Dian and surrounded by mountains to the north, west, and east, and the altitude of downtown is 1891 meters. Kunming belongs to the subtropical monsoon climate, and the average temperature is around 16.5°C. The annual precipitation is about 1450 mm, belonging to high humidity area. Besides, Kunming is a major tourist and trade city, with the GDP being 4300 billion yuan in 2016. With the rapid development of Kunming, the environment problems need to be paid more attention.

Yuxi is located in the center of Yunnan Province, about 90 kilometers south of Kunming. It is located between north latitude of 23°19′ and 24°53′N and east longitude of 101°16′ and 103°09′E. Like many of the central and eastern parts of the province, it is part of the Yunnan-Guizhou Plateau. The area is 15,285 km^2^ and the population is approximately 2.5 million. Tempered by the low latitude and moderate elevation, Yuxi has a mild subtropical highland climate, with short, mild, dry winters, and warm, rainy summers. The annual average temperature is about 15.4–24.2°C and the precipitation is about 787.8–1000 mm. In addition to the complex nature conditions, Yuxi is an important economy center and its GDP is 1309 billion yuan in 2016, so it plays a pivotal role in the development of Yunnan Province.

### 3.2. Data Description

**Figure 4:**
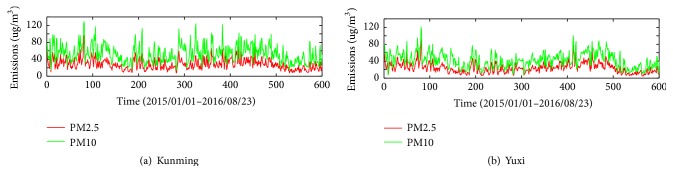
The original PM_2.5_ and PM_10_ of Kunming and Yuxi.

The data sets of daily PM_2.5_ and PM_10_ concentration of Kunming and Yuxi used in this paper are retrieved from the website of the online air quality monitoring and analyze platform of China (https://www.aqistudy.cn/historydata/). The daily PM_2.5_ and PM_10_ concentrations data from January 1, 2015, to August 23, 2016, in Kunming and Yuxi are collected (Figure 4). Each data set is divided into two sets: the training data set including 491 data points (from January 1, 2015, to May 5, 2016) and the remaining 110 data points as the testing data set (from May 6, 2016, to August 23, 2016).

Table 1 shows the statistics of the training, testing, and total data for daily PM_2.5_ and PM_10_ concentrations of Kunming and Yuxi. The recorded daily maximum PM_2.5_ is 95.8 and 91 for Kunming and Yuxi, both appearing on March 22, 2015. The recorded daily maximum PM_10_ is 129 and 121 for Kunming and Yuxi, appearing on July 9, 2016, and June 19, 2015, respectively.

### 3.3. Evaluation Criteria

The root-mean-square error (RMSE), the mean absolute error (MAE), the mean bias error (MBE), and Pearson's correlation coefficient (*r*) are used to evaluate the reliability of CI-FPA-SVM model. RMSE and MAE measure residual errors, which give a global idea of the difference between the observed and forecast values. RMSE is used to measure the sensitivity and extremum effect of the predicted value. MAE is used to evaluate the absolute error range of the predicted value. *r* is collected to show linear correlation between observed data and forecasted value. The lower values of MAE and RMSE indicate that the model is better. MBE indicates whether the model is over- or underpredicted in general. MBE is better when it is close to 0 while *r* is better when it is close to 1. RMSE, MAE, MBE, and *r* are calculated as follows:

(8) RMSE=∑i=1nyi−y^i2n,MAE=∑i=1nyi−y^in,MBE=∑i=1nyi−y^in,r=∑i=1nyi−y−y^i−y^−∑i=1nyi−y−∑i=1ny^i−y^−,

where *y*_*i*_ is the observed value and ${\hat{y}}_{i}$ is the forecasted value to *y*_*i*_. *n* is the number of the observations of the validation set. $\overset{-}{y}$ and $\overset{-}{\hat{y}}$ stand for the mean of observed value and the mean of forecasted value, respectively.

### 3.4. Process of Cointegration Test

#### 3.4.1. Result of Unit Root Tests

To estimate the cointegration of the time series variables, all of the time variables need to be stationary in order to avoid problems with spurious correlation. The Augmented Dicky-Fuller (ADF) unit root tests are employed to test the stationarity of the time series variables being investigated in this study.  Table 2 shows the results of the ADF tests and the results indicate that all the time series variables are stationary at 0.01 significance level. Therefore, all the time series variables are regarded as cointegrated of order zero, that is, *I*(0).

#### 3.4.2. Result of Cointegration Test

Table 3 obtained from the Johansen cointegration test shows that four variables, Kunming_PM2.5, Kunming_PM10, Yuxi_PM2.5, and Yuxi_PM10 are cointegrated as indicated by the star wherein the value of trace statistic is smaller than 1% critical value. The results of the trace test and the maximum eigenvalue test verify the presence of long run relationship between Kunming_PM2.5, Kunming_PM10, Yuxi_PM2.5, and Yuxi_PM10.

### 3.5. Results and Analysis

#### 3.5.1. Results of the Proposed Model

In this study, the input consisted of the particular matter of past two days in Kunming and Yuxi. The following day's particular matter of Kunming and Yuxi is, respectively, selected as the output of the new hybrid model.

**Figure 5:**
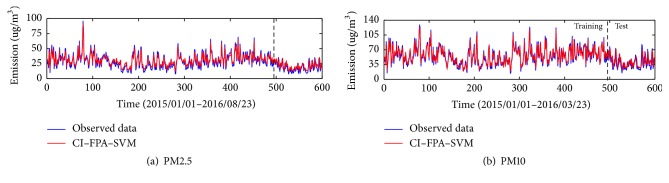
The predictive results of particular matter in Kunming.

**Figure 6:**
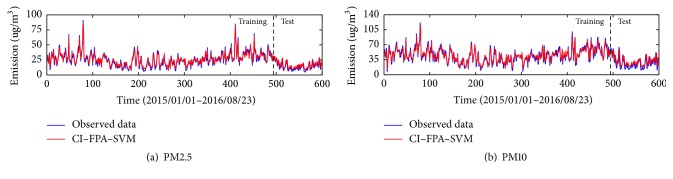
The predictive results of particular matter in Yuxi.

From Figures 5 and 6, the prediction of PM_2.5_ and PM_10_ of Kunming and Yuxi has a great performance by the proposed model. According to the results in testing periods, it can be observed that the performance of the proposed model for PM_2.5_ in Yuxi is better than that in Kunming. For PM_2.5_ forecasting, the hybrid model CI-FPA-SVM obtains the RMSE, MAE, and MBE of 6.58, 5.31, and −2.57 in Kunming, respectively, and yields the RMSE, MAE, and MBE of 4.96, 4.06, and −2.22 in Yuxi, respectively (Table 4). Meanwhile, the accuracy of PM_10_ in Kunming is higher than that in Yuxi. And the proposed model of PM_10_ achieves the RMSE, MAE, and MBE of 7.86, 6.6, and −3.22 in Kunming while the values of RMSE, MAE, and MBE are 10.35, 8.37, and −3.32 in Yuxi (Table 5). Moreover, it appears that the hybrid model CI-FPA-SVM can provide a highly accurate prediction to 1-day ahead PM time series for Kunming and Yuxi.

#### 3.5.2. Model Comparisons

In this study, the comparisons of forecasting hybrid models for the daily particular matter (PM_2.5_ and PM_10_) of Kunming and Yuxi are made among the proposed hybrid model CI-FPA-SVM (Model 1), the hybrid model CI-PSO-SVM (Model 2), the hybrid model CI-GA-SVM (Model 3), the hybrid model CI-SVM (Model 4), the hybrid model FPA-SVM (Model 5), the hybrid model CI-FPA-NN (Model 6), and multiple linear regression (Model 7). The forecasting performances of different models are presented in Tables 4 and 5. And the empirical study shows that the proposed hybrid model CI-FPA-SVM is remarkably superior to all the considered benchmark models. Furthermore, it displays that the hybrid model can combine all the advantages of each individual model.

As for forecasting of PM_2.5_ in Kunming and Yuxi in Table 4, it is apparent that the proposed hybrid model CI-FPA-SVM has a best performance among all other hybrid models. In particular, compared with CI-PSO-SVM and CI-GA-SVM, the proposed hybrid model achieves the most excellent accuracy in both two regions. And this reveals that FPA has a better optimizing performance than the traditional optimization methods (PSO and GA). What is more, it is obvious that the hybrid model FPA-SVM acquires worse predictive result in this study; this means the cointegration theory plays an important role in the hybrid model. Meanwhile, we also can draw the conclusion that the prediction of PM_2.5_ in Yuxi is superior to that in Kunming.

Next, the performance of the proposed hybrid model CI-FPA-SVM and compared models in the prediction of the PM_10_ in Kunming and Yuxi is displayed in Table 5. Firstly, overall, the proposed model has an outstanding performance in Kunming and Yuxi among all the models. Secondly, FPA can achieve two better parameters of SVM than PSO and GA by comparing CI-FPA-SVM to CI-PSO-SVM and CI-GA-SVM. Thirdly, it can be found that the three statistical errors (MAE, RMSE, and MBE) of FPA-SVM are highest in both Kunming and Yuxi, which means CI has an important role in prediction.

Then, it must be noticed that Model 6 and Model 7 are considered the classical models for PM concentration forecasting. The performance of Model 6, in which the artificial neural network is selected as the main algorithm to get nonlinear relationship between input and output, is better but is worse than proposed Model 1 according to four indicators (MAE, RMSE, MBE, and *r*). Meanwhile, Model 7, as the most traditional method to get linear relationship by least square method, has obtained the worst precise accuracy among all seven considered benchmark models.

Above all, the hybrid model CI-FPA-SVM in this paper is simple and quite efficient in the prediction of PM.

## 4. Conclusions

In order to predict the particular matter pollution, the serious environmental issues, this paper proposes a new model called CI-FPA-SVM, which combined flower pollination algorithm with support vector machine (FPA-SVM) based on cointegration theory (CI). The model consists of two parts. The prior part introduces the information related to ambient sequences into the hybrid model by cointegration theory, so it can make full use of the information for prediction. The cointegration theory provides a useful and effective tool for extracting functional relationships between inputs and outputs, and it can avoid the occurrence of spurious regression. To establish the forecasting part, SVM, in which the parameters c and g are optimized by FPA, is employed in this study. In the empirical study, the proposed hybrid model CI-FPA-SVM is utilized to forecast daily PM_2.5_ and PM_10_ concentrations in Kunming and Yuxi. Compared with six benchmark models, including FPA-SVM model which has no cointegration theory as foundation, CI-SVM model which rejects optimization algorithm, and two other models based on cointegration theory but optimized by traditional algorithms, GA and PSO, called CI-PSO-SVM and CI-GA-SVM, and two classical methods, CI-FPA-NN model and multiple linear model, the results indicate that the proposed hybrid model CI-FPA-SVM is remarkably superior to all considered benchmark models in both Kunming and Yuxi, in terms of its higher predictive accuracy.

However, in this paper, we only take the correlation of particular matters (PM_2.5_ and PM_10_) and the influence of the surrounding city into consideration, without considering the possible impacts of other pollutants, such as NO, CO_2_, and SO_2_. It is obvious that the factors are important for prediction. Investigating how to probe into appropriate and reasonable components to construct the model may be a future research direction. As mentioned above, an interesting potential direction would be the use of this novel hybrid model to further enhance and optimize the performance.

## Figures and Tables

**Table 1:** Statistical parameters of PM_2.5_ and PM_10_ in each data set.

Particular matter	Region	Min	Max	Mean	Std	SK	CV
PM2.5	Kunming						
	All	7.70	95.80	28.34	12.33	0.98	0.43
	Training	7.70	95.80	30.45	12.26	0.95	0.40
	Test	7.80	35.50	18.95	7.20	0.55	0.38
	Yuxi						
	All	4.80	91.00	24.20	12.06	1.23	0.50
	Training	5.90	91.00	26.28	12.00	1.25	0.46
	Test	4.80	36.00	14.92	6.91	0.79	0.46

PM10	Kunming						
	All	14.00	129.00	53.12	20.72	0.69	0.39
	Training	14.00	129.00	56.16	20.66	0.63	0.37
	Test	15.00	82.50	39.55	14.74	0.89	0.37
	Yuxi						
	All	6.80	121.00	42.18	17.85	0.52	0.42
	Training	6.80	121.00	45.19	17.27	0.51	0.38
	Test	10.30	64.80	28.76	13.81	0.85	0.48

Min: the minimum. Max: the maximum. Std: the standard deviation. SK: the skewness. CV: the coefficient of variation.

**Table 2:** The ADF unit root test on time series.

	Kunming_PM2.5	Kunming_PM10	Yuxi_PM2.5	Yuxi_PM10
*t*-statistic	−11.35	−11.87	−4.46	−9.80
Prob.	0.00	0.00	0.00	0.00

**Table 3:** The unrestricted cointegration rank test on four time series.

	Hypothesized number of CE(s)	Eigenvalue	Trace statistic	0.05 critical value	Prob.
Unrestricted cointegration rank test (trace)	None	0.11	216.72	47.86	0.00
	At most 1	0.11	144.37	29.80	0.00
	At most 2	0.07	76.86	15.49	0.00
	At most 3	0.06	35.31	3.84	0.00

Unrestricted cointegration rank test (maximum eigenvalue)	None	0.11	72.34	27.58	0.00
	At most 1	0.11	67.51	21.13	0.00
	At most 2	0.07	41.55	14.26	0.00
	At most 3	0.06	35.31	3.84	0.00

**Table 4:** The results of predicting PM_2.5_ for seven models.

Region	Indicator	Model						
		1	2	3	4	5	6	7
Kunming	MAE	5.31	5.34	5.78	8.61	10.59	5.56	13.71
	RMSE	6.58	6.57	6.97	10.06	12.07	6.79	16.98
	MBE	−2.57	−2.72	−3.12	−7.64	−10.67	−2.86	−0.46
	*r*	0.83	0.81	0.79	0.71	0.63	0.80	0.58

Yuxi	MAE	4.06	4.32	5.6	6.6	9.18	4.48	13.04
	RMSE	4.96	5.21	6.95	7.86	10.44	4.99	15.81
	MBE	−2.22	−2.55	−3.45	−5.47	−8.66	−2.61	−0.59
	*r*	0.84	0.81	0.78	0.75	0.67	0.81	0.61

**Table 5:** The results of predicting PM_10_ for seven models.

Region	Indicator	Model						
		1	2	3	4	5	6	7
Kunming	MAE	6.6	9.18	10.56	10.57	10.93	8.69	14.02
	RMSE	7.86	10.44	13.23	12.98	13.41	11.03	18.63
	MBE	−3.22	−3.32	−3.65	−5.47	−8.66	−3.46	−1.29
	*r*	0.86	0.83	0.81	0.68	0.64	0.82	0.62

Yuxi	MAE	8.37	8.59	8.64	9.62	9.76	8.61	14.06
	RMSE	10.35	10.60	10.65	12.01	12.08	10.63	18.93
	MBE	−3.71	−3.84	−3.97	−4.28	−4.92	−3.89	−1.64
	*r*	0.85	0.82	0.79	0.75	0.71	0.81	0.68

## Abbreviations

ADF: Augment Dickey-Fuller
AIC: Akaike information criterion
ANN: Artificial neural network
ARIMA: Autoregressive integrated moving average model
CCEMD: Complementary ensemble empirical mode decomposition
CI: Cointegration theory
FPA: Flower pollination algorithm
GA: Genetic algorithm
GWO: Grey wolf optimizer
ICCEMD: Improved ensemble empirical mode decomposition
MAE: Mean absolute error
MBE: Mean bias error
OLS: Ordinary least square
PM: Particular matter
PSO: Particle swarm optimization
RMSE: Root-mean-square error
SC: Schwartz criterion
SI: Swarm intelligence
SVM: Support vector machine
SVR: Support vector regression
WOA: Whale optimization algorithm
RBF: Radial basis function.
